# Supplementary material for: Prenatal developmental toxicity of Urtica simensis essential oil in rat embryos and rat fetuses
Source: PLoS One. 2025 Jul 31;20(7):e0329486. doi: 10.1371/journal.pone.0329486 (PMC12312955; doi:10.1371/journal.pone.0329486)
Supplement: Attachment — Submitted filename: Authors Response to Editor and Reviewer Comments.docx [file pone.0329486.s003.docx]

**Editor Comments:**

Dear Author,

Thank you for submitting your manuscript entitled “Developmental and Reproductive Toxicity of Urtica simensis Essential Oil in Pregnant Rats.” The study addresses an important public health concern related to the traditional use of herbal remedies during pregnancy and provides valuable preclinical data. Upon editorial review, several strengths were noted, including adherence to OECD guidelines, appropriate animal handling, and comprehensive assessment of embryonic and fetal outcomes. However, to enhance the scientific rigor, translational relevance, and clarity of the manuscript, the following comments and recommendations are provided for your consideration. Overall significance and originality

Dear editor, Thank you so much for your thorough review of our manuscript and valuable comments, which improve the quality of the manuscript. Dear editor, we have addressed your comments and suggestions accordingly. The revisions are indicated by track changes in the revised manuscript.

**Comments and suggestions:**

**A. Background and methodology**

**1**. While the study adds valuable insight into potential risks, the novelty is modest unless the authors provide more extensive chemical profiling and mechanistic interpretation.

Dear editor, Thank you. We have done GC-MS analysis for identification of compounds in the essential oil.

**2.** Exposure: The translational relevance to human exposure remains somewhat speculative and would benefit from contextual pharmacokinetic bridging (e.g., comparative doses, route of exposure relevance).

- Why were oral gavage chosen over inhalation or dermal exposure, which may better reflect traditional use (e.g., body fumigation)? The oral route is reasonable for worst-case systemic exposure, but this rationale should be articulated.

Dear editor, Thank you. Oral studies are prioritized for hazard identification because they establish a clear dose-response relationship for systemic effects. The oral route guarantees the highest level of absorption when compared to inhalation or dermal routes, which may have lower bioavailability due to obstacles such as skin or lung clearance. In addition, many regulatory frameworks mandate that oral toxicity data be obtained before assessing alternative exposure routes. Dear Editor, however, if strong evidence emerges supporting inhalation or dermal exposure, further studies may be warranted in the future.

**3**. Use of 5000 mg/kg Limit Dose in Acute Toxicity

- OECD Guideline 425 allows the use of 5000 mg/kg as a limit test only when no prior evidence suggests the substance is toxic. However, essential oils often contain bioactive or even cytotoxic compounds (e.g., eugenol, 1,8-cineole, aromatic hydrocarbons), which are mentioned by the authors themselves. There’s no chemical safety data presented upfront to reassure that 5000 mg/kg is a biologically plausible or ethical starting point.

- Editorial Suggestion: The authors should:

• Briefly cite prior toxicity reports or justify absence of toxicity warnings from traditional or preclinical data.

• Consider whether lower limit doses (e.g., 2000 mg/kg) would have been more conservative and aligned with OECD recommendations for substances of unknown or expected toxicity.

Dear editor, thank you. As noted in OECD 425, the use of a limit dose (5000 mg/kg) is exceptional and should only be applied when scientifically justified—particularly when the findings have direct relevance to human/animal health/environmental safety. In our case, the test substance is a traditional nutraceutical herb widely consumed in Ethiopia (mentioned in the introduction). Dear Editor, given its history of nutraceutical use, we determined that a high-dose assessment was critical for evaluating potential risks under extreme exposure scenarios. Thus, in such conditions, the 5000 mg/kg starting dose did not violate animal ethics or animal welfare.

**4.** Areas for Improvement:

• The GC-MS chemical profile should be summarized in the current manuscript (even if previously published) to contextualize the toxicological relevance.

Dear editor, thank you. We have summarized the chemical profiles of the essential oil (Table 1).

• Clarify if the dosing mimics potential human exposure levels (oral vs. inhalation/topical).

Dear editor, Thank you. As we have explained in the above comment, the most common exposure to the plant material was oral for therapeutic purposes such as diabetes and cardiac disease and for nutritional purposes like juice, tea, and stew.

• Histological assessment is reported as “no abnormalities,” but quantification or scoring of histological parameters would enhance objectivity.

Dear editor, thank you. We have included tabulated histopathological scores for each placenta. But we did not find a placenta with pathological lesion (Table 9).

**B. Results and Interpretation**

The results are clearly presented with detailed tables and figures. There is a dose-dependent effect, particularly at 1000 mg/kg, suggesting developmental toxicity.

• The absence of malformations with fetal growth restriction and resorption increase implies embryolethality, not necessarily teratogenicity. This distinction should be clarified.

Dear editor, thank you very much. Embryolethality means toxicity to the embryo, even death, typically occurring during early gestation (often before organogenesis is complete). Teratogenicity means the induction of structural or functional abnormalities in a developing fetus (during organogenesis or later). Dear editor, as you explained, embryolethality does not mean teratogenicity, but teratogenicity can be a subset of embryolethality if no structural abnormality is present. The true distinction was the timing of gestation to say "rat embryo (0-12 days)" and "rat fetuses (13 days-birth)." Dear editor, based on the comment, we modified the title to "Prenatal Developmental Toxicity." Instead of teratogenic effects.

• The discussion occasionally overstates potential mechanisms (e.g., neurotoxicity due to aromatic hydrocarbons) without direct evidence or reference to plasma/tissue levels of these compounds.

Dear editor, Thank you. We tried to rewrite some narrations based on your comment. We discussed only reported scientific evidences, particularly about some compounds that are found in the *U. simensis* essential oil.

**C. Writing and Presentation**

The manuscript is detailed but verbose in several sections, particularly the methodology. The writing is generally clear but would benefit from professional editing to improve conciseness and scientific tone.

• Condense repetitive procedural descriptions.

• Improve sentence flow in the discussion to avoid speculative overreach.

• Use standardized toxicological terminology (e.g., “no observed adverse effect level,” “maternal toxicity,” “embryo-foetal development”).

Dear editor, Thank you. We have carefully gone through the manuscript for grammar, punctuation, and scientific tone, including standard toxicological terms. We have also addressed repetitive procedural descriptions and speculative narrations. It was also proofread by a native English speaker from the Department of English Language.

Editorial Decision: Major Revision

Required Revisions

1. Summarize the essential oil composition within the current manuscript.

2. Clarify relevance of dose levels to human exposure and routes of administration.

3. Provide a clearer distinction between embryotoxicity and teratogenicity.

4. Consider including histological scoring or more quantitative assessment of placental findings.

5. Streamline the methodology and discussion for clarity and precision.

6. Moderate speculative mechanistic interpretations unless supported by referenced data.

Dear editor, Thank you once again. We carefully addressed your major concerns accordingly.

**Reviewer #1: Dear authors,**

Thank you for being able to read this manuscript. The idea is interesting, but many points need to be changed to improve readers' understanding.

Dear reviewer, Thank you so much for the detailed review of our manuscript. Dear reviewer, we carefully addressed your comments, suggestions, and questions, which enhance the integrity and clarity of our manuscript for publication.

1) The text of the manuscript should be under extensive review of English.

Dear Reviewer, thank you. We have carefully reviewed the manuscript for English language clarity and had it proofread by a native English speaker from the Department of English Language.

2) The title does not match the manuscript. The authors did not only evaluate the teratogenic effect. They evaluated fetal and placental development.

Dear Reviewer, Thank you; we have corrected the title of the manuscript based on your comments.

3) Introduction

- There is no hypothesis. Please insert the hypothesis of this study in introduction.

Dear Reviewer, Thank you. After your comment, we have stated the hypothesis of this study in the introduction (Page 3, lines 40-41).

4) Methodology

- The phytochemical constituents of the plant were not evaluated in this study. However, numerous studies in the literature have investigated the phytochemistry of this species, revealing significant variations in their findings. These discrepancies may arise from differences in several factors, such as the plant parts analyzed (e.g., leaves, stems, or fruits), the geographic origin of the samples, the time of year they were collected, and the extraction methods employed. It is well-established that these variables can significantly influence both the types and concentrations of active principles present.

- Some information must be included, such as geographic coordinates and time of year the plant was collected.

Dear reviewer, Thank you. We have done GC-MS analysis to identify the chemical compositions of the essential oil (Page 5, lines 55-61). The geographic coordinates and time of year the plant was collected were also included based on your comment (Page 4, line 47).

- It is not clear what sample size was used. “five groups of twenty gravid rats”. Twenty rats in each group or 20 in the whole experiment? Then, how many rats were killed on day 12 and how many were left to die at the end of the pregnancy? These information must be clear.

Dear reviewer, Thank you. This experimental study used five groups, with 20 gravid rats in each group. Ten gravid rats in each group (fifty pregnant rats) were killed on day 12 of the experiment (Page 6, line 83). The remaining ten gravid rats in each group were killed in the day-20 experiment (Page 7, line 91).

- Since there was no change in the control groups (group IV – Vehicle and V -ad libitum), couldn't they be placed together and this be reported? This is a suggestion, which could improve the visualization of the results.

Dear reviewer, Thank you. According to the OECD guideline, the two control groups were principally different. Group IV (pair-fed control) received only the vehicle (distilled water with 2% Tween 80), but group V (ad libitum control) was kept untouched during the experiment—no administration of even the vehicle. Dear editor, the assumption was to separately evaluate the vehicle control and controls without manipulation—ad libitum. Thus, even though there were no changes between the two controls groups, merging the two controls violates this assumption.

- In statistical section, the analysis can be performed only by the software used in this study? If not, remove this information of statistical section. The results of the analysis must be the same regardless of the program used, or even if the calculation are done without any software.

Dear reviewer, Thank you. We only used SPSS version 25 software for analysis.

5) Results

- The results could be presented more effectively to enhance data comprehension. A) Control groups (Group I and II) should be presented first in tables and figures. B) Tables should be standardized, with groups consistently placed on the x-axis (or y-axis) across all tables. C) Sample numbers should be included in every table for reference.

Dear reviewer, Thank you. We have corrected based on your comments.

- Why was water intake not evaluated in Table 1?

Dear reviewer, Thank you very much. We were not unaware of the omission of water intake data during the initial submission. Dear reviewer, we have included in the revised submission.

- Some data could be consolidated into single tables for better organization. For example: Tables 5 and 6 could be combined into one table. Similarly, Tables 7 and 8 could be merged into a single table.

Dear reviewer, Thank you once again. For simple clarity, we have merged the tables based on your comment.
